# ALZHEIMER’S DISEASE AND RELATED DEMENTIAS DIAGNOSES IN THE US BY RACE, ETHNICITY, AND NATIVITY

**DOI:** 10.1093/geroni/igad104.1222

**Published:** 2023-12-21

**Authors:** Tiffany Kindratt, Laura Zahodne, Kristine Ajrouch, Florence Dallo

**Affiliations:** University of Texas at Arlington, Arlington, Texas, United States; University of Michigan, Ann Arbor, Michigan, United States; Eastern Michigan University, Ypsilanti, Michigan, United States; Oakland University, Rochester, Michigan, United States

## Abstract

Timely clinical diagnosis of ADRD is important for intervention, resource allocation, and mitigating safety concerns. While studies have examined ADRD diagnoses disparities by race/ethnicity, few include its intersection with nativity. Our aims were to: 1) estimate the odds of diagnosed ADRD among US- and foreign-born racial/ethnic groups compared to US-born White older adults and 2) compare US- and foreign-born older adults within each racial/ethnic group. We linked 2000-2017 National Health Interview Survey (NHIS) and 2001-2018 Medical Expenditure Panel Survey (MEPS) data (65+ years; n=38,033). Race/ethnicity and nativity were measured using NHIS data. Diagnosed ADRD was determined using ICD-9 (290/294/331/797) or ICD-10 (F01/F03/G30/G31) billing codes created from self-reported ADRD diagnoses during MEPS household interviews. Covariates were measured using MEPS data. US-born Black (OR=1.74; 95%CI=1.48-2.05), Hispanic (OR=1.62; 95%CI=1.14-2.29) and foreign-born Hispanic (OR=1.63; 95%CI=1.24-2.15) older adults had higher odds of diagnosed ADRD compared to US-born White older adults after adjusting for age and sex. Results were no longer significant among either Hispanic group after adjusting for education, health insurance, and access to care. However, after adjusting for education, health insurance, access to care, and chronic conditions, US-born Black older adults, but not foreign-born Black older adults, had 1.54 times greater odds (95%CI=1.27-1.87) of diagnosed ADRD compared to US-born White older adults. There were no statistically significant differences in ADRD diagnosis by nativity within each racial/ethnic minoritized group. Findings highlight the need for including nativity in studies comparing racial/ethnic groups to Whites to fully capture the burden of ADRD among US-born Black older adults.

